# Effects of dietary Chaihu-Shugan-San on egg production performance, egg quality, and hepatic lipid metabolism of late-laying hens

**DOI:** 10.3389/fvets.2026.1836155

**Published:** 2026-05-21

**Authors:** Zhenzhen Ji, Lei Xi, Lingli Chen, Xinlei Wang, Jieru Qin, Ailian Geng

**Affiliations:** 1 College of Animal Science and Technology, Henan University of Animal Husbandry and Economy, Zhengzhou, China; 2 Feed Division, Henan Provincial Animal Husbandry Technology Popularization Station, Zhengzhou, China; 3 Institute of Animal Husbandry and Veterinary Medicine, Beijing Academy of Agriculture and Forestry Sciences, Beijing, China

**Keywords:** Chaihu-Shugan-San, egg production performance, egg quality, laying hens, lipid metabolism

## Abstract

This study aimed to evaluate the effects of Chaihu-Shugan-San (CSS) on egg production, egg quality, ovarian development, serum lipid levels, liver function, and hepatic lipid metabolism in laying hens. A total of 480 67-week-old laying hens (Jinghong No. 1) were randomly assigned to four groups. Each group comprised eight replicates of 15 hens. A basal diet was fed to the control group (CON), and a basal diet supplemented with 0.5% (CSS1), 1.5% (CSS2), or 2.5% CSS (CSS3) was fed to the other groups, respectively. The trial lasted 8 weeks. The results showed a significant increase in the egg-laying rate of the CSS2 group by 6.80% than the CON group (*p* < 0.05). Haugh unit was 9.34%, 10.45%, and 7.97% higher in the CSS1, CSS2, and CSS3 groups than in the CON group (*p* < 0.05). Eggshell thickness of the CSS2 group was found to be 38.68% greater than the CON group (*p* < 0.05). The oviduct index was enhanced by 31.53%, 19.70%, and 26.89% in the CSS1, CSS2, and CSS3, respectively (*p* < 0.05). The hens in the CSS2 and CSS3 groups displayed a greater number of SYF, at 71.73% and 52.43% (*p* < 0.05). The serum triglyceride (TG) levels demonstrated a significant increase in the CSS2 group, with 40.15% (*p* < 0.05). The identification of 1,826 and 1,828 lipid compounds in the CON and CSS groups, respectively, was indicated by lipidomics analysis. 175 differential lipids (SDLs) were upregulated and 60 SDLs were downregulated significantly. Kyoto Encyclopedia of Genes and Genomes (KEGG) topological analysis indicated that SDLs in the liver were primarily enriched in the metabolism of glycerophospholipid, glycerolipid, and glycine, serine, and threonine. In conclusion, dietary CSS supplementation improves egg-laying rate and egg quality during the late-laying period, while reducing liver fat accumulation. Dietary level of 1.5% is recommend. The reason may be due to its effects on hepatic lipid profile and metabolism, especially through glycerophospholipid and glyceride metabolism pathways.

## 1 Introduction

In line with the recent advances in modern breeding techniques and the refinement of production systems, the poultry industry has proposed extending the laying period for commercially flocked hens to 100 weeks old or longer (1). As laying hens transition from their peak egg-laying period to the mid-to-late stage, their performance gradually declines, resulting in deteriorating eggshell quality and feed conversion efficiency. These factors have a direct impact on the profitability of the poultry industry (2). The underlying mechanisms are intricate and multifaceted, comprising physiological impairments such as diminished ovarian function, reduced reproductive hormone secretion, enhanced oxidative stress, reduced immune function, and disturbed gut microflora (3, 4). Consequently, the identification of safe and efficacious intervention strategies to alleviate this aging-related physical decline is crucial for preserving the productive capability.

Traditional Chinese herbal medicine (CHM) has alleviated the suffering of countless patients with its distinctive theory and treatment methods, sparking the interest of researchers. Its increasing popularity is due to its many advantages, including that it leaves no residue, is low in toxicity, is widely available, and inexpensive (5, 6). In light of both the urgent need to extend the laying cycle and of enhance egg production performance, traditional CHM offers a potential solution.

Chaihu-Shugan-San (CSS) is a traditional compound Chinese herbal medicine. It was first published in the medical classic Jingyue Quanshu during the Ming Dynasty (7). The CSS formula consists of *Bupleurum falcatum L., Paeonia lactiflora Pall, Ligusticum chuanxiong Hort, Citrus reticulata Blanco, Glycyrrhiza uralensis Fisch, Citrus aurantium L., and Cyperus rotundus L*. As we known there have no published reports on the application of CSS in poultry, but most of the components of CSS have been studied and applied in poultry. Dietary supplementation of *Bupleurum falcatum L*. saikosaponins alleviated the inhibitory effects of ammonia exposure on the growth performance of broiler chickens (8). Dietary supplementation of paeoniflorin enhanced the antioxidant capacity of broiler chickens (9). *Ligusticum* chuanxiong extract promoted preovulatory follicles angiogenesis in laying hens and improved ovarian function in older laying hens (10). Ju et al. (11) reported that dietary addition of Chenpi can enhance the antioxidant capacity of yellow-feathered broiler chickens, lower blood lipid levels, and improve their health. Dietary addition of *Glycyrrhiza uralensis* extract can improve the growth performance of broiler chickens and increase the number of beneficial bacteria in the cecum (12). *Citrus aurantium L*. var. *dulcis* peel essential oil can reduced intestinal damage caused by coccidiosis in broiler chickens and improved their growth performance (13). *Cyperus rotundus* tubers powder can improved the meat quality and antioxidant properties of broiler chickens (14). The utilization of CSS has been documented in both pre-clinical (15) and clinical (16) contexts for the management of depression, with evidence supporting its safety and efficacy. In China, the CSS has been used for many years to treating liver diseases due to its soothing effects on the liver, its regulation of qi, promotion of blood circulation, and relief of pain (17, 18). The liver of laying hens synthesis 90% of the body's fat from scratch, and is involved in synthesizing 84% of egg yolk precursors (19). It also synthesizes 85%−90% of systemic proteins (20). High-yielding laying hens must sustain peak egg production (laying rate >90%) for nearly 40 weeks, resulting in prolonged high-load operation of the liver. The liver of laying hens is more susceptible to damage than other organs or tissues, which subsequently impacts production performance (21). Previous researches have evidenced the efficiency of CSS in reducing lipid accumulation and exhibiting potent anti-inflammatory properties (7, 22, 23). Feng et al. (24) found that saikosaponin reduced liver cholesterol accumulation and alleviated fatty liver in laying hens through microbiota-BA-intestinal FXR crosstalk. However, the effect of dietary CSS on reducing fat in laying hens and its subsequent impact on egg production remains unclear.

The aim of this study was to investigate the effects of CSS on hepatic lipid metabolism and egg-laying performance in laying hens at the late laying period. To this end, we evaluated production performance, serum biochemical parameters, liver histopathology, and elucidated potential metabolic pathways through liver lipidomics.

## 2 Materials and methods

### 2.1 Preparation of CSS

The CSS used in this trial was supplied by Henan Yuzhou Weihua Herbs Store (Henan, China). The medicinal materials were then ground into a fine powder, sieved through a 40-mesh screen, thoroughly mixed in the specified proportions, and stored at 25 °C for future use. The constituents of CSS are as follows per 100 g: *Bupleurum falcatum L*. 15.3 g, *Paeonia lactiflora Pall* 12.7 g, *Ligusticum chuanxiong Hort* 12.7 g, *Citrus aurantium L*. 8.5 g, *Citrus reticulata Blanco* 16.9 g, *Glycyrrhiza uralensis Fisch* 8.5 g, and *Cyperus rotundus L*. 25.4 g in the ratio 3.6:3:3:2:4:2:6. The main nutritional components of CSS are presented in Table 1.

**Table 1:** The main nutritive composition of CSS.

Ingredients	Content, %
Crude protein	9.03
Crude fat	2.1
Ash	4.8
Ca	0.63
P	0.26

### 2.2 Experimental design and management

A total of 480 67-week-old laying hens (Jinghong No. 1) from Henan De'an Agricultural Technology Co., Ltd. (Zhengzhou, China) were randomly assigned to four groups. Each group comprised eight replicates of 15 hens. A basal diet was fed to the control group (CON), and a basal diet supplemented with 0.5% (CSS1), 1.5% (CSS2), or 2.5% CSS (CSS3) was fed to the other groups, respectively. The doses of CSS were selected based on previous literature (25) and standard dose-response design principles. The trial lasted 8 weeks. The recommendation was set out in GB/T 5916-2020 (26) and was used to formulate the basal diet. The basal diet formulation and nutrient levels are shown in Table 2. The laying hens were raised in a four-tiered cage system. Each cage has 3 chickens. Cage dimensions: 40 cm × 35 cm × 38 cm. The room temperature was maintained at 23 °C ± 2 °C, and relative humidity was maintained at 55% ± 5% throughout the 8-week experimental period. During the trial, the hens were fed in four portions (at 06:00, 09:00, 14:00, and 17:00). The hens had free access to water. We set the photoperiod at 16 h of light and 8 h of darkness during the whole trial. Laying performance was analyzed at the replicate unit; serum parameters, ovarian development, liver morphology, and liver lipidomics were analyzed at the individual bird unit; and egg quality was analyzed at the replicate unit.

**Table 2:** The ingredient and nutrient composition of the basal diet (air-dry basis).

Ingredients	Content, %	Nutrient content	Content^2^
Corn	62.00	Metabolizable energy, MJ·kg^−1^	11.43
Soybean meal	26.70	Crude protein, %	17.04
Soybean oil	1.00	Calcium, %	4.93
Limestone	9.30	Available phosphorus, %	0.12
Premixes^1^	1.00	Lysine, %	0.83
Total	100.00	Methionine, %	0.31

^1^The premix provided per kilogram of diet: vitamin A 12,000 IU, vitamin D3 1,500 IU, vitamin E 25 IU, vitamin K 40 mg, vitamin B1 5 mg, vitamin B2 15 mg, vitamin B6 15 mg, vitamin B12 0.05 mg, vitamin C 186 mg, folic acid 1.8 mg, biotin 0.3 mg, choline 500 mg, Cu 3 mg, Fe 75 mg, Mn 80 mg, Zn 80 mg, I 0.8 mg, Se 0.4 mg.

^2^The nutrient levels were calculated from data provided by the Feed Database in China (2013) (54).

### 2.3 Sample collection

On the evening before the trial ends (75 weeks), 1 hen from each replicate (8 hens per treatment) is selected at random and fasted for 12 h. The blood samples (5 ml) were extracted from the wing vein and stored at ambient temperature for 2 h. Serum is obtained by centrifuging the blood sample at 3,000 × g for 10 min at 4 °C. The serum is stored at −20 °C for biochemical analysis. These hens were sacrificed by cervical dislocation. The following organ tissues were removed and weighed: liver, heart, spleen, ovaries and oviduct. The number of small yellow follicles (SYF) (6–8 mm in diameter) and large white follicles (LWF) (3–5 mm in diameter) was counted. About 0.5 cm × 1 cm × 1 cm of liver tissue was taken from a fixed sampling site and immersed in 4% paraformaldehyde for histological examination. Subsequently, 2 g of liver tissue was cut and placed in sterile enzyme-free tubes, which were stored at −80 °C for lipid metabolomics analysis.

### 2.4 Egg production performance

The egg production performance was evaluated by recording the daily egg production, egg weight, feed consumption, and number of unqualified eggs (including cracked eggs and soft-shelled eggs) during 67–75 weeks. Then, using replicates, the egg-laying rate, average egg weight, proportion of unqualified eggs, average feed intake, and feed conversion rate (total feed consumption/total egg production) were all calculated.

### 2.5 Egg quality

At the end of the trial (75 weeks), three eggs were randomly selected from each replicate. and 24 eggs in each group for egg quality assessment. The vertical diameter, horizontal diameter, and shell thickness of eggs were measured using a vernier caliper (Xinchengliang, Chengdu, China). Eggshell strength was determined using an eggshell strength tester (YN-100, Yaoen, Nanjing, China). Egg yolk color, albumen height, and the Haugh unit were all measured using an automatic egg quality tester (EMT-7300, Robotmation, Japan). Eggshell weight, as well as yolk weight, were determined using an electronic balance (WT 15000X, Wantai, Changzhou, China). The calculation formulas are as follows: egg shape index = vertical diameter/horizontal diameter, eggshell ratio = eggshell weight/egg weight, yolk ratio = yolk weight/egg weight.

### 2.6 Organ index and ovarian development

The organ indexes were calculated using the formula: organ index = fresh organ weight/live chicken weight (g/kg). The diameter of the largest follicle was measured using tape. The number of LPF and SYF was recorded.

### 2.7 Liver morphology

Liver tissue samples were obtained and put in 4% paraformaldehyde. The samples were then embedded in paraffin wax, sliced, dehydrated, and stained with haematoxylin and eosin (H&E) and Oil Red O. The stained tissues were carefully observed using a fully automatic scanning microscope (BA600-4, Motic China group Co., Ltd., Xiamen, China) (Version 1.0.8.18b). For each liver sample, five randomly selected fields per section (at 200 × magnification) were captured. The percentage of lipid droplet area relative to total tissue area was quantified using Fiji software (http://fiji.sc/).

### 2.8 Serum lipid and liver function parameters

The levels of serum total cholesterol (TC), triglycerides (TG), high-density lipoprotein (HDL), and low- density lipoprotein (LDL) were examined. So were alanine transaminase (ALT) and aspartate transaminase (AST). The examination was carried out using a fully automatic biochemical analyzer with respective detection kits.

### 2.9 Liver sample preparation for lipidomic analysis

Based on the production performance data (Table 3), the CSS2 group (1.5% CSS) showed the most significant improvement in egg production. Therefore, to elucidate the hepatic lipidomic mechanism underlying the CSS supplementation, we compared only the CON and CSS2 groups. From the CON and the CSS2 groups, four liver tissue samples were selected for lipid extraction. The samples were then thawed at room temperature and weighed precisely at 50 mg each. Subsequently, the liver sample was transferred into a 2 ml plastic microtube. Subsequently, it was amalgamated with a mixture comprising methanol (2:5, by volume) and water, along with 400 μl of MTBE. Homogenization was carried out at −1 °C using a high-throughput tissue crusher operating at 50 Hz for 6 min, after which the samples were subjected to ultrasonication at 40 kHz for 30 min at 5 °C. The homogenates were then placed at −20 °C for 30 min and centrifuged at 13,000 × g for 15 min at 4 °C. An aliquot of 350 μl from the upper lipid-containing phase was carefully transferred to a fresh tube and evaporated to dryness under a gentle nitrogen stream. For subsequent UHPLC—MS/MS analysis, the dried lipid extracts were reconstituted in 100 μl of isopropanol: acetonitrile (1:1, v/v) by brief sonication in a 5 °C water bath. The reconstituted samples were then subjected to a centrifugal process, whereby they were spun for a duration of 15 min at 13,000 × g at 4 °C, utilizing a benchtop centrifuge. Following this, the clarified upper layer of the mixed sample was transferred to autosampler vials. Finally, 2 μl of each sample was injected into the UHPLC-MS/MS system for lipidomic analysis.

**Table 3:** Effects of CSS on egg production performance of laying hens during 67–75 weeks.

Items	CON	CSS1	CSS2	CSS3	SEM	*P* value
Egg-laying rate, %	81.36^bc^	79.19^c^	86.89^a^	84.28^ab^	0.879	0.006
Average egg weight, g	62.22	62.23	60.94	61.89	0.206	0.080
Proportion of unqualified eggs, %	0.53	0.52	0.16	0.13	0.080	0.123
Average daily feed intake, g/bird/d	110.52	110.47	110.20	110.65	0.830	0.911
Feed conversion ratio, g/g	2.18	2.25	2.11	2.17	0.021	0.142

^a−*c*^In the same row, values with different letter superscripts indicate significant differences (*p* < 0.05).

### 2.10 Lipidomic data analysis, pathway analysis

The analysis of the data was conducted on MajorBio Cloud platform (cloud.majorbio.com). Lipid metabolites were classified according to the LIPID MAPS nomenclature system, and a comparative analysis was conducted on the lipid subclasses and quantities within the two groups. Partial Least Squares-Discriminant Analysis (PLS-DA) and Orthogonal Partial Least Squares Discriminant Analysis (OPLS-DA) were used to analyze the data and identify differences between samples. Subsequently, a permutation test was conducted to evaluate the predictive capability of the model. The presence of differential lipid compounds was determined through the application of predefined statistical thresholds (*p* < 0.05) and a multivariate statistical variable importance in projection (VIP) threshold (VIP > 1). A pathway analysis using KEGG topology analysis to further explore the effects of significantly differential lipids (SDLs). The *p*-value and pathway significance were evaluated using relative betweenness centrality.

### 2.11 Statistical analysis

The normal distribution of the data was determined using the Shapiro-Wilk test in SPSS 24.0 (SPSS, Chicago, Illinois), and the homogeneity of variances was assessed using one-way analysis of variance (ANOVA). Differences were compared using Duncans multiple comparisons. The data were presented as the means and SEM. *p* < 0.05 was identified as statistically significant.

## 3 Results

### 3.1 Egg production performance

The effects of different dose of dietary CSS supplementation on egg production performance of laying hens from 67 to 75 weeks of age are shown in Table 3. During 67–75 weeks, a significant increase in the egg-laying rate of the CSS2 group by 6.80% compared with the CON group (*p* < 0.05).

### 3.2 Egg quality

The effects of different dose of dietary CSS supplementation on egg quality of laying hens at 75 weeks of age are shown in Table 4. Haugh unit at 75 weeks of age was 9.34%, 10.45%, and 7.97% higher in the CSS1, CSS2, and CSS3 groups than in the CON group (*p* < 0.05). Eggshell thickness of the CSS2 group at 75 weeks of age was found to be 38.68% greater than that of the CON group (*p* < 0.05).

**Table 4:** Effects of CSS on egg quality of laying hens at 75 weeks of age.

Items	CON	CSS1	CSS2	CSS3	SEM	*p* value
Egg shape index	1.35	1.32	1.31	1.33	0.007	0.222
Eggshell strength, kg/cm^2^	0.35	0.33	0.35	0.35	0.006	0.632
Eggshell thickness, mm	2.87^b^	2.86^b^	3.98^a^	3.32^ab^	0.167	0.049
Eggshell ratio, %	12.83	12.31	13.56	12.12	0.275	0.257
Yolk ratio, %	26.69	26.24	26.79	26.64	0.285	0.916
Albumen height, mm	10.06	8.94	9.20	8.93	0.266	0.399
Yolk color	6.14	6.63	5.85	6.10	0.138	0.251
Haugh unit	87.44^b^	95.61^a^	96.58^a^	94.41^a^	1.251	0.032

^a, b^In the same row, values with different letter superscripts indicate significant differences (*p* < 0.05).

### 3.3 Organ index and ovarian development

The effects of different dose of dietary CSS supplementation on organ index and ovarian development of laying hens at 75 weeks of age are shown in Table 5. The oviduct index was significantly enhanced by 31.53%, 19.70%, and 26.89% in the CSS1, CSS2, and CSS3, respectively (*p* < 0.05). The hens in the CSS2 and CSS3 groups displayed a greater number of SYF, at 71.73% and 52.43% respectively (*p* < 0.05).

**Table 5:** Effects of CSS on organ index and ovarian development of laying hens at 75 weeks of age.

Items	CON	CSS1	CSS2	CSS3	SEM	*p* value
Organ index, g/kg						
Liver index	18.09	19.31	16.95	16.64	0.658	0.525
Heart index	4.62	4.48	4.56	4.84	0.183	0.936
Spleen index	0.93	1.00	1.25	1.30	0.083	0.341
Ovary index	24.39	25.27	20.21	22.45	0.908	0.205
Oviduct index	29.08^b^	38.25^a^	34.81^a^	36.90^a^	1.253	0.015
Follicular development						
Number of LWF	31.67	22.33	23.33	32.67	2.321	0.269
Number of SYF	7.00^b^	9.00^ab^	12.00^a^	10.67^a^	0.721	0.046
The largest follicle diameter, cm	3.13	3.37	3.13	3.43	0.090	0.582

^a, b^In the same row, values with different letter superscripts indicate significant differences (*p* < 0.05).

### 3.4 Serum lipid and liver function

The effects of different dose of dietary CSS supplementation on serum lipid and liver function of laying hens at 75 weeks of age are shown in Table 6. Compared with the CON group, serum TG levels demonstrated a significant increase in the CSS2 group with 40.15% (*p* < 0.05). Dietary CSS supplementation did not affect the ALT and AST levels of laying hens (*p* > 0.05).

**Table 6:** Effects of CSS on serum lipid and liver function of laying hens at 75 weeks of age.

Items	CON	CSS1	CSS2	CSS3	SEM	*p* value
ALT, U/L	2.79	2.85	3.05	2.93	0.469	0.998
AST, U/L	230.03	251.69	248.27	246.91	4.355	0.325
TC, mmol/L	2.83	3.05	2.62	2.92	0.183	0.897
TG, mmol/L	28.47^b^	31.06^b^	39.90^a^	31.23^b^	1.654	0.043
HDL, mmol/L	0.35	0.30	0.32	0.34	0.048	0.991
LDL, mmol/L	1.11	1.25	0.96	1.22	0.102	0.792

^a, b^In the same row, values with different letter superscripts indicate significant differences (*p* < 0.05).

### 3.5 Liver morphology

**Figure 1:**
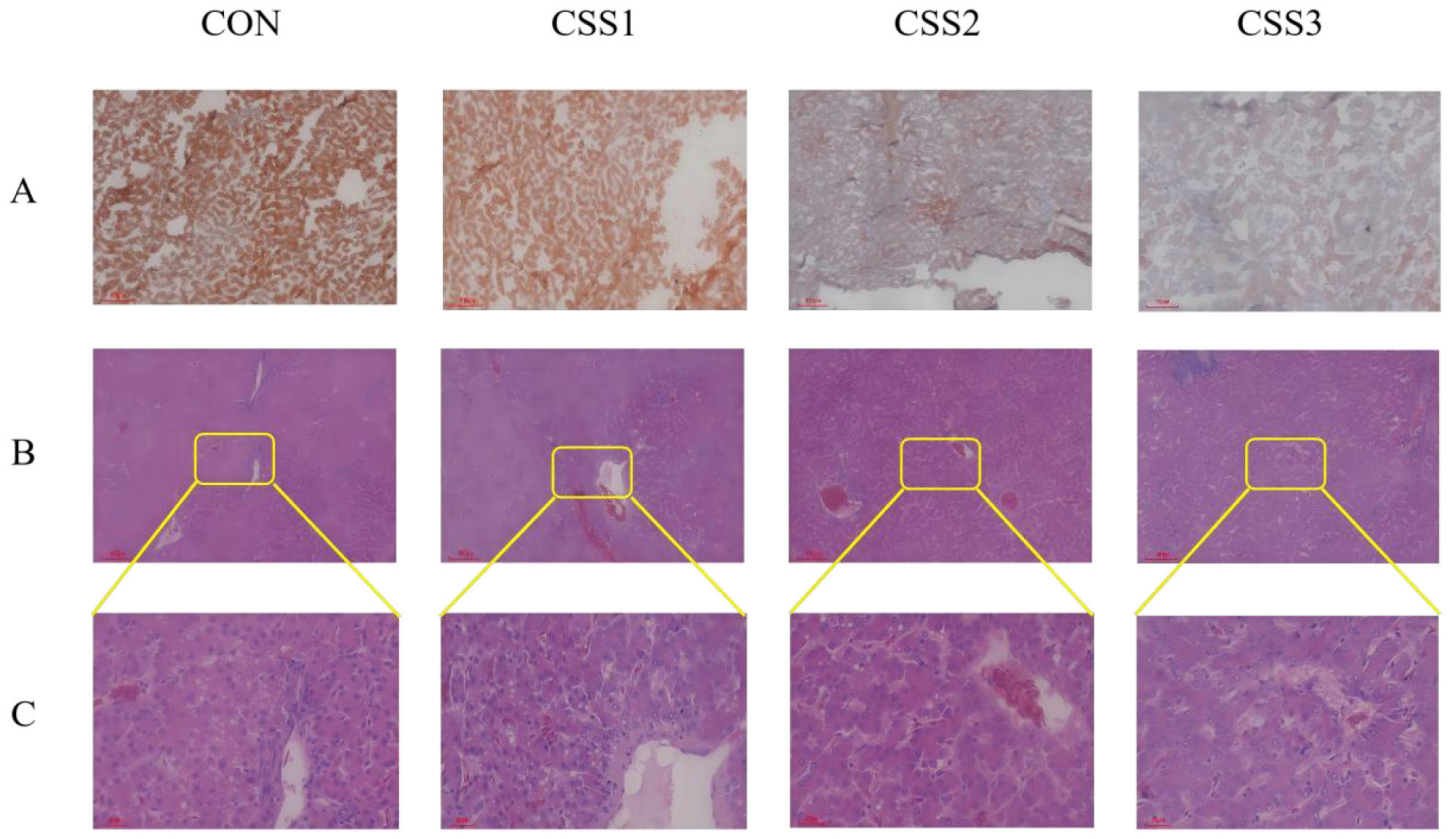
Effect of CSS on the changes in the liver morphology at 75 weeks of age. **(A)** The liver morphology was stained with Oil Red O. **(B)** The liver morphology was after H&E staining. **(C)** The inset from **(B)** is shown in an enlarged view. his is a figure. Schemes follow the same formatting.

The effects of different dose of dietary CSS supplementation on liver morphology of laying hens at 75 weeks of age are shown in Figure 1. The Oil Red O staining results showed that the CSS2 and CSS3 groups exhibited less fat accumulation (Figure 1A). At the same time, histological examination of HE stained sections revealed that the CSS supplementation reduced the number of fat vacuoles (Figures 1B, C). As shown in Table 7, the quantitative analysis results of liver tissue sections indicated that the percentage of lipid droplet area in the livers of broilers in the CSS2 and CSS3 groups was significantly lower than that in the CON group (*p* < 0.05).

**Table 7:** Effects of CSS on lipid accumulation in the liver of laying hens at 75 weeks of age.

Items	CON	CSS1	CSS2	CSS3	SEM	*p* value
Percentage of lipid droplet area with Oil Red O stained sections, %	1.05^a^	0.90^ab^	0.57^c^	0.77^bc^	0.535	0.030
Percentage of lipid droplet area with H&E stained sections, %	1.59^a^	1.18^ab^	0.87^b^	0.70^b^	0.119	0.004

^a − c^In the same row, values with different letter superscripts indicate significant differences (*p* < 0.05).

### 3.6 Hepatic lipid composition

As a result, CSS2 showed the greatest effect; livers from the CON and CSS2 groups were selected for further lipidomic studies.

**Figure 2:**
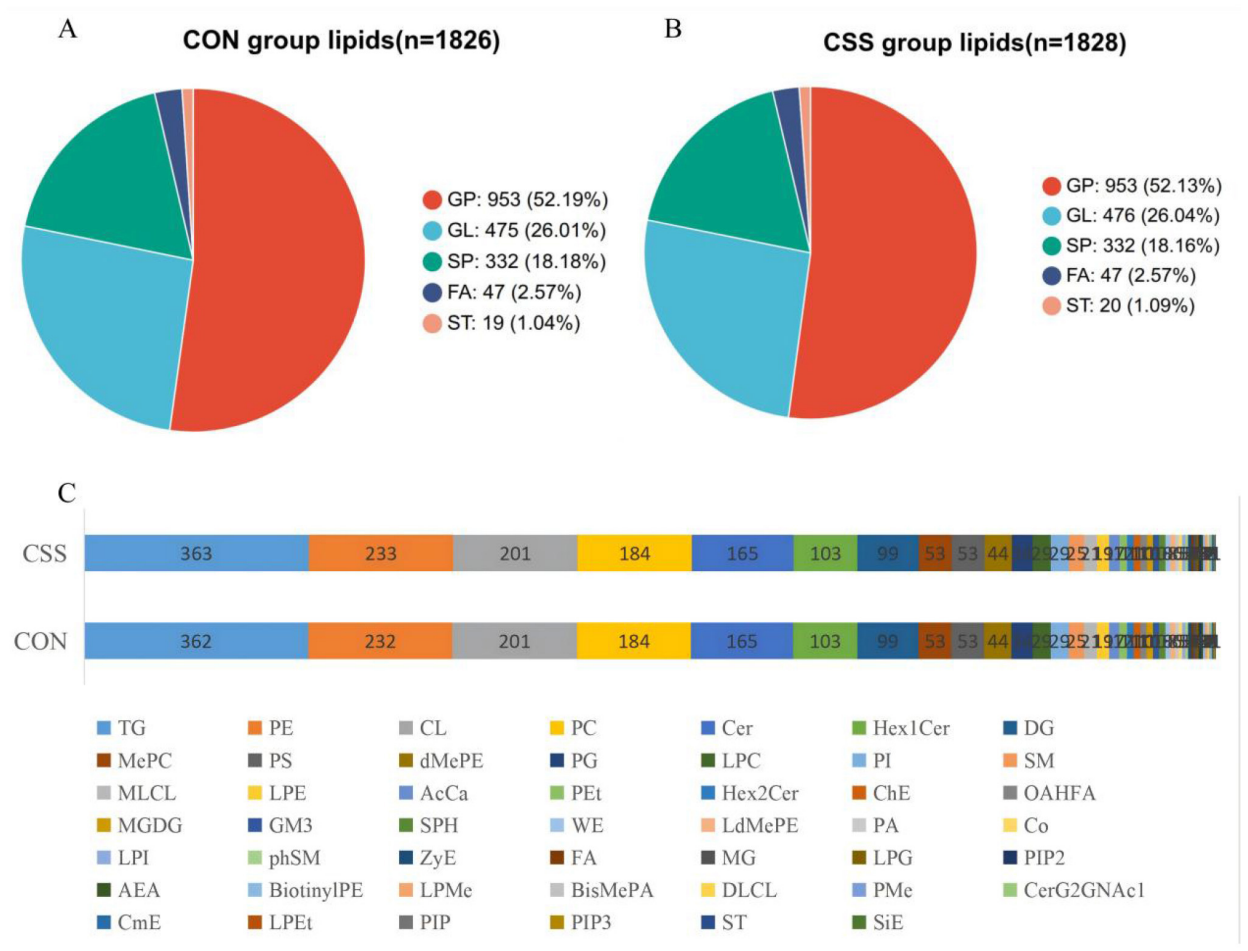
Analysis of the composition of hepatic lipids. **(A)** The pie chart of hepatic lipid composition in the CON group. **(B)** The pie chart of hepatic lipid composition in the CSS group. **(C)** The bar chart comparing lipid number in lipid subclasses of the CON and CSS groups. CSS, basal diet supplemented with 1.5% Chaihu-shugan-San; *n*, the total number of lipid molecules in this group.

The lipid metabolite categories identified by the CON group and CSS group, along with the number of lipid species in each category, were shown in Figures 2A, B. The comparison of lipids in each subclass between the CON group and the CSS group (Figure 2C). Overall, there were no significant differences between the two groups with regard to lipid categories or numbers.

### 3.7 Hepatic lipid profile

**Figure 3:**
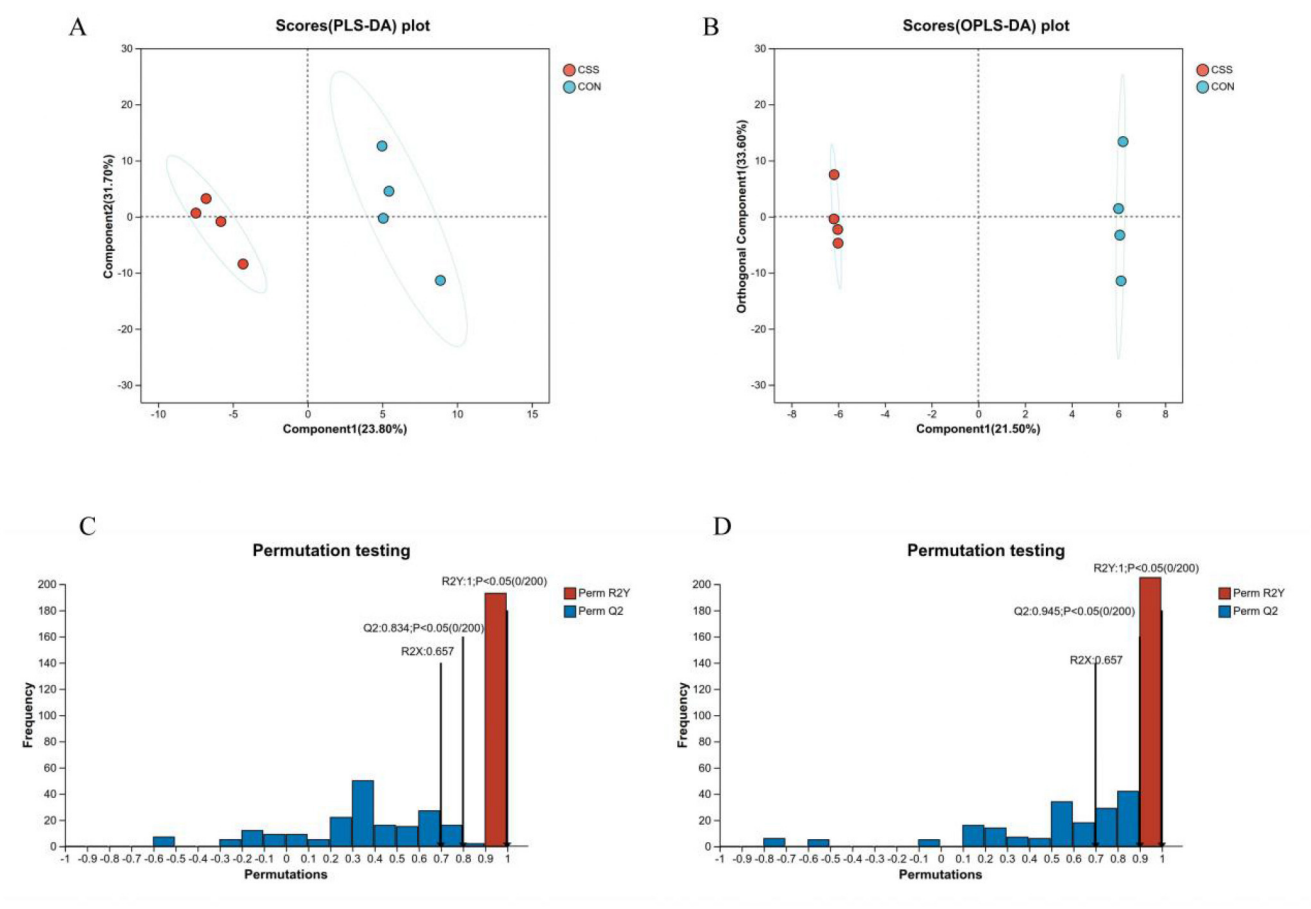
Analysis of the profile of hepatic lipids. **(A, B)** Liver lipidome PLS-DA and OPLS-DA scores. **(C, D)** PLS-DA and OPLS-DA permutation test. CSS, basal diet supplemented with 1.5% Chaihu-shugan-San.

To analyse the separation of the CON and CSS groups and distinguish differences in lipid profiles, PLS-DA (Figure 3A) and OPLS-DA (Figure 3B) were performed on liver samples. The results showed that the liver samples from the CSS group were significantly distant from CON group samples (*p* < 0.05). The generated PLS-DA model exhibited the following goodness-of-fit and predictive capability parameters: *R*^2^*X* = 0.657, *R*^2^*Y* = 1, *Q*^2^ = 0.945. The generated OPLS-DA model demonstrated the following parameters: *R*^2^*X* = 0.657, *R*^2^*Y* = 1, *Q*^2^ = 0.834, confirming the model's fitting effect and predictive performance (Figures 3C, D), which indicates high intragroup cohesion within the CSS and CON groups, with clear intergroup separation, revealing significant metabolic differences in the lipid profiling of liver tissue between the two groups of laying hens.

### 3.8 Analysis of SDLs and metabolic pathways

**Figure 4:**
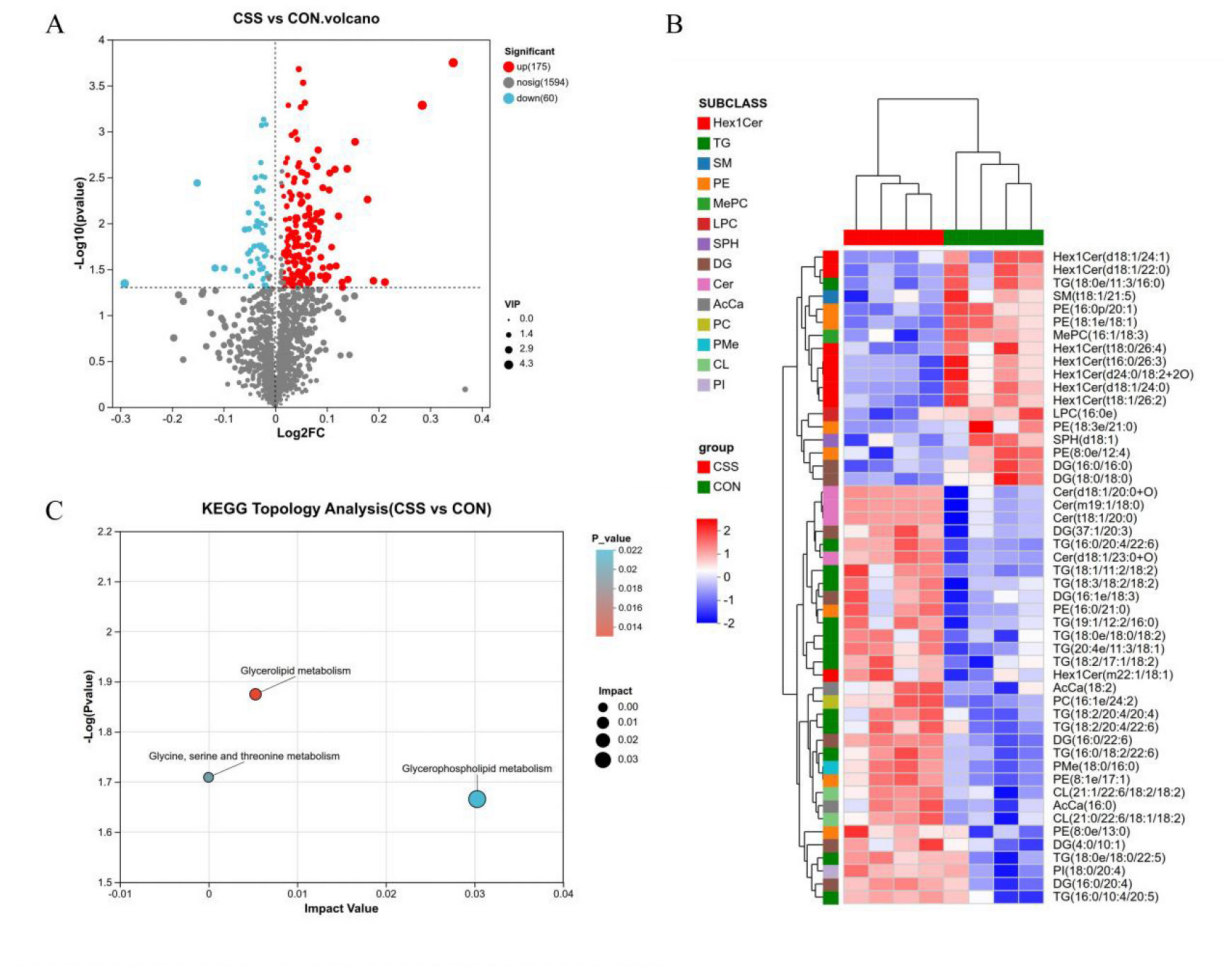
Analysis of differential lipids and metabolic pathways. **(A)** Volcano plot of differential lipid of layers in CON and CSS groups; each dot indicates a lipid species, and the size of the dot shows its significance. Nosig, no significant. **(B)** Heatmap of lipid molecule clusters with significant differences. **(C)** Differential lipids KEGG topology analysis. CSS, basal diet supplemented with 1.5% Chaihu-shugan-San.

The most significant lipids contributing to lipid profile differences were identified and visualized in a heatmap based on their trend patterns. Volcano plot analysis (Figure 4A) revealed lipid metabolites between the CSS and CON groups, including 235 SDLs with 175 that were found to be significantly upregulated and 60 significantly downregulated (Supplementary Tables S1, S2). These findings suggested that CSS induces significant changes in the composition and quantity of lipid species.

Cluster analysis was performed on lipids that showed significant differences, with the results shown in Figure 4B. Analysis of lipid subclasses revealed that the CSS group exhibited significant downregulation of the Hex1Cer and PE lipid subclasses in the liver. In contrast, the TG, DG, CL, PE, PG, and Cer lipid subclasses showed overall significant upregulation. Both up-regulation and down-regulation of PE subclass occurred. The up-regulated PE species were predominantly those containing polyunsaturated fatty acids (PUFAs), such as PE (16:0/18:3), which contains α-linolenic acid, as well as PE (17:0/22:6) and PE (15:0/22:6), which contain DHA. Conversely, the down-regulated PE species were those containing long-chain saturated or monounsaturated fatty acids, such as PE(18:0/20:1), PE (18:1/22:0) and PE (22:0/20:4), as well as ether-linked PE and plasmalogen species, such as PE (18:1e/18:1), PE (16:0e/16:0) and PEt (16:0/18:1) (Supplementary Tables S1, S2). To investigate whether hepatic lipid metabolism is affected by CSS, 235 SDLs were mapped to the KEGG database. In total, 235 SDLs were searched against the KEGG pathway database, resulting in the identification of 89 metabolic pathways (Supplementary Table S3). The most relevant metabolic pathways identified herein were glycerophospholipid, glycerolipid, and glycine, serine, and threonine metabolism (Figure 4C). Out of all these, the glycerophospholipid metabolism pathway turned out to be the most significantly relevant, while the glycerolipid metabolism pathway was the second most relevant.

## 4 Discussion

High-performance liquid chromatography/mass spectrometry (HPLC/MS) analysis found that CSS contains 12 primary compounds, including tangeretin, hesperetin, α-cyperone, saikosaponin A, saikosaponin D, paeonin, liquiritin, glycyrrhizic acid ammonium salt, naringin, hesperidin, neohesperidin, and ferulic acid (27). Network pharmacology methods identified that CSS contains 730 compounds and 917 corresponding target genes, and that CSS exerts its pharmacological effects through multiple targets (28).

As we know, this is the first study to use CSS in laying hens for enhancing egg production performance. The laying rate, feed efficiency, and egg quality are the primary economic indicators and directly reflect the hen's overall physiological state. Xiao et al. (29) showed that treatment with a mixture of daidzein and CHM (*Astragalus membranaceus, Salvia miltiorrhiza* Bunge, and *Cnidium monnieri*) elevated the egg-laying rate of Hyline Brown hens by 2.8% and the average egg weight by 1.6 g. Similarly, another study found that adding 1% CHM mixture consisting of 70% *pine needle* and 30% *Artemisia annua* significantly increased the egg production rate and had the lowest feed -to-egg ratio (30). Yu et al. (31) demonstrated that dietary 0.8% CHM (comprising *Epimedium, Astragalus, Angelica, Leonurus* and *Houttuynia*) can significantly increase the average egg weight of Jinghong No. 1 laying hens in the late laying stage. Jiang et al. (32) found that dietary 2% fermented and enzymatically fermented CHM (comprising *Astragalus, Chinese hawthorn, Wolfiporia extensa Ginns, Angelica sinensis, Leonurus japonicus, Alpinia oxyphylla*, and dandelion) significantly increased egg production rates while evaluating feed efficiency of late-laying Hy-Line Brown hens.This suggests that CHM can enhance the egg production when given as a dietary supplement. In this trial, we observed that dietary CSS had a beneficial effect on laying rate during 67–75 weeks, which may be due to its various nutrients, including proteins, lipids, minerals and vitamins (33). Maintaining nutritional balance and enhancing growth performance were key functions performed by these nutrients (30).

As laying hens transition from their peak laying period to the late laying stage, their physiological functions decline, and their ability to digest and absorb nutrients also decreases. This leads to a decline in egg production and quality (1, 34). Previous studies found that dietary CHM improved Haugh unit (fermented S. *chinensis* pomace, fermented pine needle extract, and Chinese chive powder) (35) and shell strength of Hyline Brown hens (daidzein, *Astragalus membranaceus, Salvia miltiorrhiza* Bunge, and *Cnidium monnieri*) (29). Dietary CHM (*Epimedium, Astragalus, Angelica, Leonurus*, and *Houttuynia*) was found to increase eggshell strength, albumen height, and Haugh unit in Jinghong No. 1 laying hens (31). Liu et al. (36) also found that Dietary CHM significantly improved the concentration of docosatetraenoic acid (C20:4n-6) in egg yolk of Wenchang breeder hens. In this study, Haugh unit of CSS supplementation was improved, likely due to CSS's powerful antioxidant properties, which are effective in preventing protein break down by reducing protein oxidation (37).

During follicular development in laying hens, a series of dominant follicles form and mature sequentially, ultimately producing eggs for ovulation. During this process, the liver of laying hens synthesizes 90% of the body's lipids (38). The liver's health status is closely related follicle numbers and egg-laying rate. This dietary study observed that dietary CSS increased the oviduct index and the number of SYF, consistent with earlier results showing improved egg production rate (29–31).

The process of egg formation requires substantial lipid deposition (39). Lipid metabolism is reflected by TC, TG, HDL, and LDL. At the same time, the levels of ALT and AST are generally assessed to indicate whether cardiac and hepatic cells function in animals has been impaired (40). Previous findings have mainly indicated that Chinese herbal medicine and ginger powder can lower serum TC and TG levels of Lohmann layers under heat stress (41); adding 0.25%, 0.50%, and 1.00% herbal additives (*forsythia, scrophularia*, and *andrographis*) to the diet reduced blood TC and TG levels in Donglan Black Chickens (42). In this study, a significant elevation in serum TG levels was observed among subjects in the CSS2 group. High serum TG is generally considered a risk factor for cardiovascular disease, but this does not apply to high-yield egg-laying hens. In laying hens, circulating TG are not merely pathological markers; they serve as essential precursors for yolk formation. The hen's liver synthesizes large quantities of TG, which are packaged into very-low-density lipoproteins (VLDL) and transported to the developing ovarian follicles (43). Therefore, a moderate increase in serum TG may reflect enhanced hepatic VLDL export and efficient lipid allocation toward egg production. Hepatic lipid homeostasis depends not only on synthesis rates but also on the efficiency of lipid efflux mediated by VLDL (44). However, in this study, histological analysis revealed that CSS led to a reduction in lipid accumulation within the liver. Collectively, these findings suggest that CSS2 (1.5%) promoted the redistribution of hepatic lipids to the bloodstream for ovarian uptake, which is further supported by the concurrently improved egg production rate in the CSS2 group. In nonalcoholic fatty liver rat models, the CSS can reduce the accumulation of lipids in the liver caused due to a high-fat diet and improve impaired liver function (27, 45). The present study extends this observation by demonstrating that in laying hens—a species with high physiological lipid demand—the liberated hepatic lipids are redirected toward reproductive output. As shown by Zheng et al. (45), the presence of certain active components, such as quercetin and linolenic acid, within CSS has been found to regulate microRNAs, thereby increasing the biosynthesis of fatty acids. Ren et al. (27) showed that naringenin is likely the active compound in CSS and that silent information regulator 1 (SIRT1) is the hub gene through which CSS mediates treatment of non-alcoholic steatohepatitis. Importantly, serum ALT and AST levels remained within normal ranges across all treatments, thus, elevated liver and serum TG levels did not result in accumulation within the liver. The TG may therefore provide an adequate source of lipids for sustained follicle development and eggshell formation. The co-occurrence of Cer upregulation and Hex1Cer downregulation suggests that CSS does not simply cause ceramide accumulation but may specifically regulate ceramide metabolic flux. Tong et al. (46) indicated that ceramides of specific chain lengths act as crucial signaling molecules that regulate energy metabolism and potentially redistribute energy via specific signaling pathways (e.g., AMPK), thereby prioritizing the requirements of egg production. Another study showed that CSS-containing serum significantly inhibited the decrease in mitochondrial membrane potential caused by corticosterone, also increasing the oxygen consumption rate of both maximum and reserve mitochondrial respiration, which may be related to mitochondrial dynamics and energy metabolism mediated by the PGC-1α/SIRT1 signaling pathway (47). CL, a phospholipid unique to the inner mitochondrial membrane, is crucial for maintaining mitochondrial structure and function. It does this by stabilizing the electron transport chain and facilitating oxidative phosphorylation (48, 49).

PE is a diverse family of glycerophospholipids whose species differ in fatty acyl chain composition, and these species are not functionally interchangeable (50). PE rich in polyunsaturated fatty acids help enhance membrane fluidity (51), whereas PE rich in saturated and monounsaturated fatty acids primarily maintain membrane structural integrity and participate in lipid storage (52). This functional difference explains why they are regulated differently under CSS treatment. Glycine, serine, and threonine serve as crucial one-carbon unit sources for PE and PC synthesis (53). The KEGG enrichment analysis supports that glycerophospholipid metabolism and glycerolipid metabolism pathways were significantly enriched, indicating the central role of PE metabolism in CSS's regulatory effect on hepatic lipid homeostasis. Activation of this pathway may significantly enhance the assembly efficiency of membrane phospholipid and VLDL, thereby connecting the lipid metabolism network and jointly increasing egg production.

In summary, CSS may enhance egg production in late-laying hens by potentially modulating hepatic lipid metabolism. CSS supplementation is associated with a reshaping of the glycerophospholipid profile and an upregulation of CL. These changes may indicate enhanced VLDL assembly and secretion. Importantly, serum TG levels increased while hepatic lipid droplets decreased, and ALT and AST levels remained normal, suggesting a shift from hepatic lipid storage to lipid export rather than liver damage.

## 5 Conclusions

This study suggests that dietary CSS supplementation improves egg-laying rate and egg quality during the late-laying period, while reducing liver fat accumulation. Dietary level of 1.5% is recommended in this present study. The reason may be due to its effects on hepatic lipid profile and metabolism, especially through glycerophospholipid and glyceride metabolism pathways.

## Data Availability

The datasets presented in this study can be found in online repositories. The names of the repository/repositories and accession number(s) can be found in the article/supplementary material.

## References

[1] Alfonso-Carrillo C Benavides-Reyes C de Los Mozos J Dominguez-Gasca N Sanchez-Rodríguez E Garcia-Ruiz AI . Relationship between bone quality, egg production and eggshell quality in laying hens at the end of an extended production cycle (105 weeks). Animals. (2021) 11:623. doi: 10.3390/ani11030623 33652961 PMC7996911

[2] Tang W Li J Liang M Zhang J Tang T Sun X . Dietary supplementation with Enterococcus faecium Ef026 elicits a systemic improvement in Hy-line brown laying hens during the mid-to-late laying phase. Pout Sci. (2026) 105:106453. doi: 10.1016/j.psj.2026.106453 PMC12870834 41610601

[3] Wang Y Zhang C Chen X Zheng A Liu G Ren Y . Dietary supplementation of compound probiotics to improve performance, egg quality, biochemical parameters and intestinal morphology of laying hens. Front Vet Sci. (2024) 11:1505151. doi: 10.3389/fvets.2024.1505151 39776595 PMC11703898

[4] Abd El-Hack M El-Saadony M Shafi M Qattan S Batiha G Khafaga A . Probiotics in poultry feed: a comprehensive review. J Anim Physiol Anim Nutr. (2020) 104:1835–50. doi: 10.1111/jpn.13454 32996177

[5] Liu M Chen R Wang T Ding Y Zhang Y Huang G . Dietary Chinese herbal mixture supplementation improves production performance by regulating reproductive hormones, antioxidant capacity, immunity, and intestinal health of broiler breeders. Poult Sci. (2024) 103:103201. doi: 10.1016/j.psj.2023.103201 37980727 PMC10692728

[6] Yan X Yu J Liu C. Effects of different levels of Chinese herbal medicine additives on performance, serum biochemistry and antioxidant capacity of laying hens. China Feed. (2024) 19:39–42. doi: 10.15906/j.cnki.cn11-2975/s.20211908

[7] Fan Q Liu Y Sheng L Lv S Yang L Zhang Z . Chaihu-Shugan-San inhibits neuroinflammation in the treatment of post-stroke depression through the JAK/STAT3-GSK3β/PTEN/Akt pathway. Biomed Pharmacother. (2023) 160:114385. doi: 10.1016/j.biopha.2023.114385 36774722

[8] Shu G Xu D Ran C Yin L Lin J Fu H . Protective effect of dietary supplementation of Bupleurum falcatum L saikosaponins on ammonia exposure-induced ileum injury in broilers. Poult Sci. (2021) 100:100803. doi: 10.1016/j.psj.2020.10.057 33516464 PMC7936159

[9] Zhou Y Qiu C Zhou Z Zhang D Cai Y Yuan J . Influence of paeoniflorin dietary supplementation on growth performance, antioxidant status, blood parameters, carcass characteristics and meat quality in broiler chickens. Vet Anim Sci. (2025) 28:100450. doi: 10.1016/j.vas.2025.100450 40256759 PMC12008132

[10] Chen H Chen X Ping Z Fang L Jiang X Ge M . Ligusticum chuanxiong promotes the angiogenesis of preovulatory follicles (F1-F3) in late-phase laying hens. Poult Sci. (2023) 102:102430. doi: 10.1016/j.psj.2022.102430 36621100 PMC9841292

[11] Ju Y Qi L Hu Y Huang L Li L Luo Y . Effects of graded levels Citri Reticulatae Pericarpium (Chenpi) on growth performance, serum biochemical indices, meat quality, and caecal microbiota and metabolite in yellow-feathered broilers. Anim Sci J. (2025) 96:e70025. doi: 10.1111/asj.70025 39791280

[12] Lu J Li S Wu J Chen Y Jiang S Zhang G. Effect of Glycyrrhiza uralensis extract, Lactobacillus acidophilus and their combined supplementation on production performance, immunity, antioxidation and intestinal health in broilers. Front Vet Sci. (2025) 12:1675593. doi: 10.3389/fvets.2025.1675593 41180229 PMC12575101

[13] Guerrini A Zago M Avallon G Brigandì E Tedesco D. A field study on Citrus aurantium L. var dulcis peel essential oil and Yucca schidigerasaponins efficacy on broiler chickens health and growth performance during coccidiosis infection in rural free-range breeding system. Livest Sci. (2024) 282:105437. doi: 10.1016/j.livsci.2024.105437

[14] Ali HAM Zangana BSR Abdullah IH. Effect of adding different levels of Cyperus rotundus tubers powder to the diet on the physical, chemical and oxidation traits of broiler carcasses. IOP Conf Ser Earth Environ Sci. (2023) 1262:072046. doi: 10.1088/1755-1315/1262/7/072046

[15] Kim S Han J Seog D Chung J Kim N Hong Park Y . Antidepressant effect of Chaihu-Shugan-San extract and its constituents in rat models of depression. Life Sci. (2005) 76:1297–306. doi: 10.1016/j.lfs.2004.10.022 15642599

[16] Wang Y Fan R Huang X. Meta-analysis of the clinical effectiveness of traditional Chinese medicine formula Chaihu-Shugan-San in depression. J Ethnopharmacol. (2012) 141:571–7. doi: 10.1016/j.jep.2011.08.079 21933701

[17] Wang X Liu X Wang Y Yang K Yeertai Y Jia Q . Chaihu Shugan powder inhibits interstitial cells of cajal mitophagy through USP30 in the treatment of functional dyspepsia. J Ethnopharmacol. (2024) 323:117695. doi: 10.1016/j.jep.2023.117695 38163556

[18] Liang Z Pi D Zhen J Yan H Zheng C Liang Chen J . The AMPK-mTOR pathway is inhibited by Chaihu Shugan powder, which relieves nonalcoholic steatohepatitis by suppressing autophagic ferroptosis. Mediators Inflamm. (2024) 2024:4777789. doi: 10.1155/2024/4777789 39502754 PMC11535263

[19] Li J Leghari I He B Zeng W Mi Y Zhang C. Estrogen stimulates expression of chicken hepatic vitellogenin II and very low-density apolipoprotein II through ER-α. Theriogenology. (2014) 82:517–24. doi: 10.1016/j.theriogenology.2014.05.003 24938798

[20] Trefts E Gannon M Wasserman D. The liver. Curr Biol. (2017) 27:R1147–51. doi: 10.1016/j.cub.2017.09.019 29112863 PMC5897118

[21] Luo S. Effects and mechanisms of taurine remissionliver injury of hens in the late laying period (Master's thesis). Huazhong Agricultural University, Wuhan (2023).

[22] Li L Yu A Wang Z Chen K Zheng W Zhou J . Chaihu-Shugan-San and absorbed meranzin hydrate induce anti-atherosclerosis and behavioral improvements in high-fat diet ApoE-/- mice via anti-inflammatory and BDNF-TrkB pathway. Biomed Pharmacother. (2019) 115:108893. doi: 10.1016/j.biopha.2019.108893 31022598

[23] Li W Zhou R Zheng J Sun B Jin X Hong M . Chaihu-Shugan-San ameliorates tumor growth in prostate cancer promoted by depression via modulating sphingolipid and glycerinphospholipid metabolism. Front Pharmacol. (2022) 13:1011450. doi: 10.3389/fphar.2022.1011450 36545317 PMC9760688

[24] Feng J Ma H Yue Y Wang L Hao K Zhang Y . Saikosaponin a ameliorates diet-induced fatty liver via regulating intestinal microbiota and bile acid profile in laying hens. Poult Sci. (2023) 102:103155. doi: 10.1016/j.psj.2023.103155 37871490 PMC10598744

[25] Wang Y Liu Y Liu T Xiao C Wu H. The effects of compound Chinese herbal additives on ovarian and yolk development in green-shelled laying hens (in Chinese). Jiangxi J Anim Husbandry Vet Med. (2023) 02:21–4. doi: 10.3969/j.issn.1004-2342.2023.02.007

[26] State Administration for Market Regulation of China and State Administration for Standardization of China. Formula feeds for layers and broilers (GB/T 5916-2020) (2020).

[27] Ren Y Xiao K Lu Y Chen W Li L Zhao J. Deciphering the mechanism of Chaihu Shugan San in the treatment of nonalcoholic steatohepatitis using network pharmacology and molecular docking. J Pharm Pharmacol. (2024) 76:1521–33. doi: 10.1093/jpp/rgae103 39250725

[28] Nie H Deng Y Zheng C Pan M Xie J Zhang Y . Network pharmacology-based approach to explore the effects of Chaihu Shugan powder on a non-alcoholic fatty liver rat model through nuclear receptors. J Cell Mol Med. (2020) 24:5168–84. doi: 10.1111/jcmm.15166 32189432 PMC7205817

[29] Xiao Y Shao D Sheng Z Wang Q Shi S. A mixture of daidzein and Chinese herbs increases egg production and eggshell strength as well as blood plasma Ca, P, antioxidative enzymes, and luteinizing hormone levels in post-peak, brown laying hens. Poul Sci. (2019) 98:3298–303. doi: 10.3382/ps/pez178 30993323

[30] Li X He W Wang Z Xu T. Effects of Chinese herbal mixture on performance, egg quality and blood biochemical parameters of laying hens. J Anim Physiol Anim Nutr. (2016) 100:1041–9. doi: 10.1111/jpn.12473 27079126

[31] Yu A Wang M Chen L Long C Guo Y Sheng X . Effects of dietary pretreated Chinese herbal medicine supplementation on production performance, egg quality, uterine histopathological changes, and antioxidant capacity in late-phase laying hens. Front Physiol. (2023) 14:1110301. doi: 10.3389/fphys.2023.1110301 36744028 PMC9895833

[32] Jiang M Zhang T Wang Q Ge J Sun L Li M . Effects of enzymolysis and fermentation of Chinese herbal medicines on serum component, egg production, and hormone receptor expression in laying hens. Anim Biosci. (2024) 37:95–104. doi: 10.5713/ab.23.0146 37905322 PMC10766462

[33] Fan C Jin H Wu L Zhang Y Ye R Zhang W . An exploration of traditional chinese medicinal plants with anti-inflammatory activities. Evid Based Complement Alternat Med. (2017) 2017:1231820. doi: 10.1155/2017/1231820 28473862 PMC5394394

[34] Saleh A Ahmed E Ebeid T. The impact of phytoestrogen source supplementation on reproductive performance, plasma profile, yolk fatty acids and antioxidative status in aged laying hens. Reprod Domest Anim. (2019) 54:846–54. doi: 10.1111/rda.13432 30916364

[35] Moon S Lee S Lee W Niu K Hwang W Oh J . Effect of dietary supplementation of a phytogenic blend containing Schisandra chinensis, Pinus densiflora, and Allium tuberosum on productivity, egg quality, and health parameters in laying hens. Anim Biosci. (2021) 34:285–94. doi: 10.5713/ajas.20.0552 33171027 PMC7876724

[36] Liu M Huang J Ma M Huang G Zhang Y Ding Y . Effects of dietary Chinese herbal mixtures on productive performance, egg quality, immune status, caecal and offspring meconial microbiota of Wenchang breeder hens. Front Vet Sci. (2023) 10:1320469. doi: 10.3389/fvets.2023.1320469 38162476 PMC10755868

[37] Zhang L Zhong G Gu W Yin N Chen L Shi S. Dietary supplementation with daidzein and Chinese herbs, independently and combined, improves laying performance, egg quality and plasma hormone levels of post-peak laying hens. Poul Sci. (2021) 100:101115. doi: 10.1016/j.psj.2021.101115 PMC8131741 33975040

[38] Hamid H Zhang J Li W Liu C Li M Zhao L . Ma Q. Interactions between the cecal microbiota and non-alcoholic steatohepatitis using laying hens as the model. Poult Sci. (2019) 98:2509–21. doi: 10.3382/ps/pey596 30690636

[39] Burley R Evans A Pearson J. Molecular aspects of the synthesis and deposition of hens' egg yolk with special reference to low density lipoprotein. Poult Sci. (1993) 72:850–5. doi: 10.3382/ps.0720850 8502607

[40] Wen K Zhang K Gao W Bai S Wang J Song W . Effects of stevia extract on production performance, serum biochemistry, antioxidant capacity, and gut health of laying hens. Poult Sci. (2024) 103:103188. doi: 10.1016/j.psj.2023.103188 37980742 PMC10665936

[41] Ibtisham F Nawab A Niu Y Wang Z Wu J Xiao M . The effect of ginger powder and Chinese herbal medicine on production performance, serum metabolites and antioxidant status of laying hens under heat-stress condition. J Therm Biol. (2019) 81:20–4. doi: 10.1016/j.jtherbio.2019.02.002 30975419

[42] Yang C Wu Z Qin L Yan J Liao Y Wang F . Effects of Chinese herbal medicine additives on blood biochemical, immune and antioxidant properties of Donglan Black chickens (in Chinese). Heilongjiang Anim Sci Vet Med. (2023) 7:14–118. doi: 10.13881/j.cnki.hljxmsy.2022.03.0277

[43] Walzem RL Hansen RJ Williams DL Hamilton RL. Estrogen induction of VLDLy assembly in egg-laying hens. J Nutr. (1999) 129:467S−72S. doi: 10.1093/jn/129.2.467S 10064311

[44] Zhang S Li G He L Wang F Gao M Dai T . Sphingosine kinase 2 deficiency impairs VLDL secretion by inhibiting mTORC2 phosphorylation and activating chaperone-mediated autophagy. Cell Death Differ. (2025) 32:1886–99. doi: 10.1038/s41418-025-01507-6 40200091 PMC12500862

[45] Zheng C Nie H Pan M Fan W Pi D Liang Z . Chaihu Shugan powder influences nonalcoholic fatty liver disease in rats in remodeling microRNAome and decreasing fatty acid synthesis. J Ethnopharmacology. (2024) 318:116967. doi: 10.1016/j.jep.2023.116967 37506783

[46] Tong F Fu S Shen Y Hou X Liu M Wang Z . Semi-quantitative lipidomics reveals the characteristics of lipid metabolism in sheep milk fermentation. Food Res Int. (2025) 211:116517. doi: 10.1016/j.foodres.2025.116517 40356153

[47] Zhang L Zheng Q Shi J Wang P Lu J Shen J. Protective mechanism of Chaihu Shugan San against CORT-induced damage in PC12 cells based on mitochondrial dynamics (in Chinese). Zhongguo Zhong Yao Za Zhi. (2025) 50:4546–54. doi: 10.19540/j.cnki.cjcmm.20250423.401 41084471

[48] Dong J Ye F Lin J He H Song Z. The metabolism and function of phospholipids in mitochondria. Mitochondrial Commun. (2023) 1:2–12. doi: 10.1016/j.mitoco.2022.10.002

[49] Zhang H Jiang Y. Biological characteristics of cardiolipin in cardiovascular diseases (in Chinese). Chin J Biochem Mol Biol. (2025) 41:678–86. doi: 10.13865/j.cnki.cjbmb.2025.03.1325

[50] Valentine WJ Shimizu T Shindou H. Lysophospholipid acyltransferases orchestrate the compositional diversity of phospholipids. Biochimie. (2023) 215:24–33. doi: 10.1016/j.biochi.2023.08.012 37611890

[51] Harayama T Antonny B. Beyond fluidity: the role of lipid unsaturation in membrane function. Cold Spring Harb Perspect Biol. (2023) 15:a041409. doi: 10.1101/cshperspect.a041409 37277191 PMC10317061

[52] Li Z Agellon LB Allen TM Umeda M Jewell L Mason A . The ratio of phosphatidylcholine to phosphatidylethanolamine influences membrane integrity and steatohepatitis. Cell Metab. (2006) 3:321–31. doi: 10.1016/j.cmet.2006.03.007 16679290

[53] Locasale JW. Serine, glycine and one-carbon units: cancer metabolism in full circle. Nat Rev Cancer. (2013) 13:572–83. doi: 10.1038/nrc3557 23822983 PMC3806315

[54] Feed Database in China. Tables of Feed Composition and Nutritive Values in China. Beijing: Feed Database in China (2013).

