# Supplementary material for: Effects of dietary Chaihu-Shugan-San on egg production performance, egg quality, and hepatic lipid metabolism of late-laying hens
Source: Front Vet Sci. 2026 May 21;13:1836155. doi: 10.3389/fvets.2026.1836155 (PMC13233366; doi:10.3389/fvets.2026.1836155)
Supplement: Supplementary file 1 [file Table_1.docx]

Supplementary Material

TABLE S1 Lipid that significantly down-regulated in liver (compared with CON group)

| Lipids | Fold Change | P value | VIP | Lipid class |
| --- | --- | --- | --- | --- |
| Cer(d18:0/24:4) | 0.960 | 0.011700 | 2.0844 | Cer |
| Cer(d18:0/14:0) | 0.988 | 0.046750 | 1.0401 | Cer |
| Cer(m22:1/18:0) | 0.981 | 0.018460 | 1.3295 | Cer |
| Cer(d16:0/18:3) | 0.974 | 0.010760 | 1.4788 | Cer |
| ChE(18:3) | 0.968 | 0.019740 | 1.6053 | ChE |
| ChE(16:0) | 0.961 | 0.037920 | 1.6927 | ChE |
| CL(24:0/14:0/16:0/16:1) | 0.980 | 0.031880 | 1.4101 | CL |
| CL(23:0/15:0/16:0/18:1) | 0.989 | 0.019980 | 1.0084 | CL |
| CL(18:3/20:4/22:4/22:6) | 0.985 | 0.022830 | 1.1205 | CL |
| DG(16:0/16:0) | 0.984 | 0.004391 | 1.2855 | DG |
| DG(18:0/18:0) | 0.965 | 0.011530 | 1.7879 | DG |
| Hex1Cer(d18:1/24:1) | 0.989 | 0.049380 | 1.0552 | Hex1Cer |
| Hex1Cer(d18:1/20:0) | 0.978 | 0.017430 | 1.5015 | Hex1Cer |
| Hex1Cer(d18:1/25:0) | 0.980 | 0.027640 | 1.4069 | Hex1Cer |
| Hex1Cer(d18:0/16:0) | 0.978 | 0.009885 | 1.4713 | Hex1Cer |
| Hex1Cer(d18:0/18:1) | 0.985 | 0.039560 | 1.1773 | Hex1Cer |
| Hex1Cer(d20:1/18:1) | 0.983 | 0.031510 | 1.2164 | Hex1Cer |
| Hex1Cer(t20:0/19:0) | 0.976 | 0.004482 | 1.6069 | Hex1Cer |
| Hex1Cer(t20:0/18:2) | 0.984 | 0.030150 | 1.2266 | Hex1Cer |
| Hex1Cer(d18:1/26:0) | 0.976 | 0.009461 | 1.4959 | Hex1Cer |
| Hex1Cer(d18:1/24:0) | 0.981 | 0.002184 | 1.4303 | Hex1Cer |
| Hex1Cer(t18:1/26:2) | 0.986 | 0.003153 | 1.2278 | Hex1Cer |
| Hex1Cer(d18:1/22:0) | 0.983 | 0.011900 | 1.3069 | Hex1Cer |
| Hex1Cer(t18:0/26:4) | 0.987 | 0.010470 | 1.1216 | Hex1Cer |
| Hex1Cer(t16:0/26:3) | 0.984 | 0.012680 | 1.2796 | Hex1Cer |
| Hex1Cer(d18:1/18:0) | 0.967 | 0.025820 | 1.5738 | Hex1Cer |
| Hex1Cer(d24:0/18:2+2O) | 0.984 | 0.012680 | 1.2796 | Hex1Cer |
| Hex1Cer(t20:0/25:3) | 0.972 | 0.017730 | 1.5169 | Hex1Cer |
| Hex2Cer(d18:1/16:0) | 0.934 | 0.030900 | 2.2389 | Hex2Cer |
| LPC(16:0e) | 0.986 | 0.018230 | 1.1563 | LPC |
| LPC(18:3e) | 0.976 | 0.024340 | 1.3409 | LPC |
| LPE(16:1e) | 0.976 | 0.006104 | 1.6504 | LPE |
| LPE(18:1e) | 0.988 | 0.022330 | 1.1366 | LPE |
| MePC(16:1/18:3) | 0.984 | 0.007775 | 1.3569 | MePC |
| MePC(22:6/21:1) | 0.976 | 0.010590 | 1.4144 | MePC |
| MGDG(18:0e/18:1) | 0.964 | 0.021370 | 1.7818 | MGDG |
| PC(18:1e/18:1) | 0.982 | 0.006665 | 1.4461 | PC |
| PC(16:1/14:0) | 0.981 | 0.009952 | 1.4241 | PC |
| PC(18:1e/18:2) | 0.988 | 0.048850 | 1.0398 | PC |
| PC(18:2e/18:1) | 0.989 | 0.034940 | 1.0097 | PC |
| PC(20:2/22:6) | 0.983 | 0.041060 | 1.1384 | PC |
| PE(18:0/20:1) | 0.922 | 0.030740 | 2.8316 | PE |
| PE(18:1e/18:1) | 0.985 | 0.000739 | 1.4763 | PE |
| PE(22:0/20:4) | 0.988 | 0.000837 | 1.1726 | PE |
| PE(18:1/22:0) | 0.974 | 0.003185 | 1.6790 | PE |
| PE(8:0e/12:4) | 0.968 | 0.048210 | 1.6388 | PE |
| PE(16:0p/20:1) | 0.982 | 0.003073 | 1.3978 | PE |
| PE(18:3e/21:0) | 0.985 | 0.046630 | 1.0949 | PE |
| PE(8:0e/11:1) | 0.901 | 0.003636 | 2.8539 | PE |
| PE(16:0e/16:0) | 0.984 | 0.019270 | 1.1801 | PE |
| PE(12:1e/23:0) | 0.951 | 0.032700 | 1.8949 | PE |
| PE(18:3e/24:2) | 0.979 | 0.009854 | 1.3539 | PE |
| PEt(16:0/18:1) | 0.817 | 0.045220 | 3.8019 | PEt |
| PS(20:3e/18:1) | 0.982 | 0.000857 | 1.5272 | PS |
| SM(t18:1/21:5) | 0.987 | 0.047280 | 1.0194 | SM |
| SM(t18:0/24:2) | 0.965 | 0.007661 | 1.6776 | SM |
| SPH(d18:1) | 0.970 | 0.034670 | 1.5741 | SPH |
| TG(18:0e/11:3/16:0) | 0.978 | 0.010870 | 1.4581 | TG |
| TG(18:0/17:0/22:6) | 0.979 | 0.004086 | 1.4065 | TG |
| TG(20:3e/18:1/20:1) | 0.982 | 0.017230 | 1.2239 | TG |

TABLE S2 Lipid that significantly up-regulated in liver (compared with CON group)

| Lipids | Fold Change | P value | VIP | Lipid class |
| --- | --- | --- | --- | --- |
| AcCa(16:1) | 1.025 | 0.014440 | 1.4826 | AcCa |
| AcCa(16:0) | 1.026 | 0.012610 | 1.5621 | AcCa |
| AcCa(18:2) | 1.033 | 0.033780 | 1.6776 | AcCa |
| AcCa(22:6) | 1.045 | 0.029800 | 1.8354 | AcCa |
| AcCa(18:3) | 1.045 | 0.045660 | 1.7514 | AcCa |
| AcCa(20:2) | 1.051 | 0.039400 | 1.8695 | AcCa |
| BiotinylPE(10:1/18:2) | 1.025 | 0.041550 | 1.4228 | BiotinylPE |
| BiotinylPE(10:0/18:2) | 1.012 | 0.022110 | 1.0549 | BiotinylPE |
| Cer(d18:1/20:0+O) | 1.014 | 0.009123 | 1.2694 | Cer |
| Cer(d18:1/23:0+O) | 1.022 | 0.001095 | 1.687 | Cer |
| Cer(d18:1/21:0+O) | 1.026 | 0.003229 | 1.7736 | Cer |
| Cer(t18:1/24:2) | 1.023 | 0.012660 | 1.5663 | Cer |
| Cer(t18:0/18:1) | 1.026 | 0.048410 | 1.5459 | Cer |
| Cer(t18:1/16:0) | 1.030 | 0.008597 | 1.7872 | Cer |
| Cer(t18:1/22:1) | 1.019 | 0.020330 | 1.3444 | Cer |
| Cer(d18:1/18:0+O) | 1.030 | 0.035180 | 1.6652 | Cer |
| Cer(t18:1/23:1) | 1.024 | 0.026170 | 1.4832 | Cer |
| Cer(m19:1/18:0) | 1.016 | 0.006581 | 1.3807 | Cer |
| Cer(d18:1/16:0+O) | 1.033 | 0.004859 | 1.8768 | Cer |
| Cer(m19:1/16:0) | 1.026 | 0.048410 | 1.5459 | Cer |
| Cer(m17:1/24:2) | 1.023 | 0.012660 | 1.5663 | Cer |
| Cer(t18:1/20:0) | 1.016 | 0.006461 | 1.3819 | Cer |
| Cer(d18:1/21:1) | 1.028 | 0.009557 | 1.5532 | Cer |
| Cer(d18:1/16:1) | 1.034 | 0.004928 | 1.7096 | Cer |
| Cer(d18:1/18:2+O) | 1.026 | 0.014900 | 1.3779 | Cer |
| Cer(t18:0/20:0+O) | 1.020 | 0.008870 | 1.3099 | Cer |
| Cer(t18:0/16:1) | 1.034 | 0.004928 | 1.7096 | Cer |
| Cer(t18:0/23:0+O) | 1.021 | 0.005496 | 1.4054 | Cer |
| CL(19:0/22:5/18:2/16:0) | 1.025 | 0.003573 | 1.7111 | CL |
| CL(21:0/20:4/18:1/16:0) | 1.016 | 0.019080 | 1.2749 | CL |
| CL(18:2/16:0/16:0/20:1) | 1.024 | 0.013360 | 1.5856 | CL |
| CL(20:5/18:2/18:1/18:2) | 1.049 | 0.009248 | 2.2207 | CL |
| CL(21:0/22:6/18:1/18:2) | 1.018 | 0.014590 | 1.4684 | CL |
| CL(20:5/18:2/18:2/18:2) | 1.034 | 0.047420 | 1.7764 | CL |
| CL(18:2/18:1/18:2/18:2) | 1.042 | 0.010400 | 2.1083 | CL |
| CL(23:1/20:4/20:4/20:4) | 1.014 | 0.002186 | 1.2479 | CL |
| CL(23:1/22:6/20:4/18:0) | 1.032 | 0.014560 | 1.725 | CL |
| CL(21:1/16:0/15:0/20:4) | 1.018 | 0.000519 | 1.4375 | CL |
| CL(21:1/18:2/22:6/22:6) | 1.053 | 0.011200 | 2.1421 | CL |
| CL(19:0/22:6/16:1/18:2) | 1.023 | 0.046700 | 1.4165 | CL |
| CL(24:2/18:2/16:1/22:6) | 1.011 | 0.020460 | 1.0615 | CL |
| CL(18:3/18:2/20:0/22:4) | 1.218 | 0.000518 | 4.3287 | CL |
| CL(21:1/16:0/18:2/22:3) | 1.016 | 0.044010 | 1.1902 | CL |
| CL(21:1/22:6/18:2/18:2) | 1.028 | 0.012600 | 1.8057 | CL |
| CL(18:2/18:2/22:5/18:2) | 1.048 | 0.024810 | 2.0193 | CL |
| CL(18:3/22:3/15:0/22:2) | 1.041 | 0.008362 | 1.9456 | CL |
| CL(11:0/18:0/22:5/22:6) | 1.059 | 0.001592 | 2.5663 | CL |
| CL(11:3/16:0/20:4/22:4) | 1.058 | 0.002401 | 2.5841 | CL |
| CL(18:2/18:1/18:1/18:2) | 1.048 | 0.010350 | 2.1353 | CL |
| CL(18:2/18:1/18:1/22:1) | 1.030 | 0.016490 | 1.6006 | CL |
| CmE(8:0) | 1.079 | 0.018180 | 2.2766 | CmE |
| DG(18:0/22:6) | 1.030 | 0.001220 | 1.706 | DG |
| DG(16:0/22:6) | 1.032 | 0.000209 | 1.8429 | DG |
| DG(16:0/22:4) | 1.020 | 0.045290 | 1.2231 | DG |
| DG(16:0/20:4) | 1.017 | 0.014010 | 1.2844 | DG |
| DG(16:1e/18:3) | 1.023 | 0.048490 | 1.3483 | DG |
| DG(20:4e/18:2) | 1.028 | 0.022440 | 1.4724 | DG |
| DG(37:1/20:3) | 1.028 | 0.017550 | 1.5513 | DG |
| DG(4:0/10:1) | 1.022 | 0.035430 | 1.3614 | DG |
| DG(18:1/10:3) | 1.015 | 0.041970 | 1.0524 | DG |
| DG(16:2e/17:1) | 1.027 | 0.028890 | 1.4143 | DG |
| DG(37:0/22:6) | 1.028 | 0.010540 | 1.5392 | DG |
| dMePE(18:1/20:4) | 1.011 | 0.021670 | 1.0171 | dMePE |
| GM3(d18:1/24:1) | 1.037 | 0.005859 | 1.9648 | GM3 |
| GM3(d16:0/25:1) | 1.038 | 0.038160 | 1.8269 | GM3 |
| GM3(d18:2/18:2) | 1.030 | 0.036440 | 1.6049 | GM3 |
| GM3(d18:1/16:0) | 1.031 | 0.017650 | 1.5751 | GM3 |
| Hex1Cer(m22:1/18:1) | 1.010 | 0.039440 | 1.0296 | Hex1Cer |
| Hex1Cer(t16:0/24:0+O) | 1.019 | 0.013590 | 1.3779 | Hex1Cer |
| Hex1Cer(m17:0/25:2) | 1.016 | 0.015790 | 1.2141 | Hex1Cer |
| Hex1Cer(t20:0/20:2) | 1.015 | 0.023250 | 1.1702 | Hex1Cer |
| LPG(18:2) | 1.035 | 0.006141 | 1.9449 | LPG |
| LPI(20:4) | 1.028 | 0.008630 | 1.7309 | LPI |
| LPI(18:2) | 1.036 | 0.002765 | 1.9201 | LPI |
| LPI(18:0) | 1.051 | 0.036430 | 2.1074 | LPI |
| LPI(16:0) | 1.063 | 0.023430 | 2.2905 | LPI |
| MGDG(18:0/10:3) | 1.059 | 0.001592 | 2.5663 | MGDG |
| MLCL(18:2/18:2/18:2) | 1.076 | 0.029840 | 2.552 | MLCL |
| PC(16:1e/24:2) | 1.016 | 0.001947 | 1.2924 | PC |
| PE(17:0/18:1) | 1.011 | 0.034120 | 1.0805 | PE |
| PE(16:0/18:3) | 1.029 | 0.047390 | 1.6751 | PE |
| PE(17:0/22:6) | 1.011 | 0.045640 | 1.0265 | PE |
| PE(15:0/22:6) | 1.017 | 0.011630 | 1.292 | PE |
| PE(8:0e/13:0) | 1.014 | 0.039920 | 1.0438 | PE |
| PE(4:0/16:1) | 1.019 | 0.031700 | 1.1636 | PE |
| PE(8:0e/10:0) | 1.059 | 0.007986 | 2.2533 | PE |
| PE(12:1e/8:0) | 1.076 | 0.002832 | 2.5217 | PE |
| PE(30:1/18:1) | 1.033 | 0.002213 | 1.7363 | PE |
| PE(37:0/24:0) | 1.047 | 0.044350 | 1.7768 | PE |
| PE(24:2/23:1) | 1.046 | 0.020640 | 1.8655 | PE |
| PE(35:0/24:0) | 1.051 | 0.011720 | 2.0277 | PE |
| PE(16:0/21:0) | 1.028 | 0.016530 | 1.5476 | PE |
| PE(20:4e/18:3) | 1.018 | 0.040470 | 1.1312 | PE |
| PE(8:1e/17:1) | 1.027 | 0.001030 | 1.6902 | PE |
| PE(37:0/22:1) | 1.094 | 0.049860 | 2.4123 | PE |
| PEt(17:1/10:0) | 1.062 | 0.037310 | 2.0651 | PEt |
| PG(18:1/18:2) | 1.020 | 0.004585 | 1.4942 | PG |
| PG(16:0/18:2) | 1.023 | 0.005017 | 1.6245 | PG |
| PG(18:0/20:4) | 1.031 | 0.008924 | 1.7419 | PG |
| PG(18:1/20:4) | 1.011 | 0.005069 | 1.109 | PG |
| PG(18:2/20:4) | 1.022 | 0.036250 | 1.4039 | PG |
| PG(16:0/22:6) | 1.048 | 0.009906 | 2.0863 | PG |
| PG(28:0/17:1) | 1.035 | 0.000545 | 1.8511 | PG |
| PI(16:0/18:2) | 1.046 | 0.007978 | 2.2747 | PI |
| PI(18:0/20:4) | 1.027 | 0.047470 | 1.6488 | PI |
| PI(18:0/18:2) | 1.036 | 0.017680 | 1.9536 | PI |
| PI(16:0/22:6) | 1.045 | 0.005068 | 2.1561 | PI |
| PI(16:0/18:1) | 1.047 | 0.007025 | 2.1522 | PI |
| PI(16:0/16:1) | 1.064 | 0.007559 | 2.4343 | PI |
| PI(20:4/20:4) | 1.047 | 0.006805 | 2.0238 | PI |
| PIP(18:3e/22:6) | 1.057 | 0.007800 | 2.2682 | PIP |
| PIP2(18:2/20:4) | 1.024 | 0.040000 | 1.4129 | PIP2 |
| PMe(18:0/16:0) | 1.027 | 0.001013 | 1.6911 | PMe |
| PS(18:0/20:4) | 1.057 | 0.014950 | 2.352 | PS |
| PS(16:0/22:6) | 1.063 | 0.009609 | 2.4152 | PS |
| PS(16:0/18:2) | 1.070 | 0.041080 | 2.3385 | PS |
| PS(18:1e/20:4) | 1.059 | 0.013890 | 2.33 | PS |
| PS(16:1e/18:2) | 1.102 | 0.002554 | 3.0285 | PS |
| PS(18:3e/18:2) | 1.066 | 0.004097 | 2.4366 | PS |
| PS(18:0/22:6) | 1.103 | 0.040900 | 2.7228 | PS |
| TG(18:2/17:1/18:2) | 1.016 | 0.026490 | 1.2098 | TG |
| TG(16:0/20:4/22:6) | 1.038 | 0.000295 | 1.9971 | TG |
| TG(10:0/18:2/18:2) | 1.019 | 0.011560 | 1.2522 | TG |
| TG(16:0/18:2/22:6) | 1.017 | 0.002986 | 1.3502 | TG |
| TG(18:3/18:2/18:2) | 1.032 | 0.033210 | 1.5896 | TG |
| TG(18:2/20:4/20:4) | 1.023 | 0.013870 | 1.4685 | TG |
| TG(18:3/18:2/22:6) | 1.033 | 0.043670 | 1.5688 | TG |
| TG(18:0e/18:0/22:5) | 1.037 | 0.035870 | 1.7901 | TG |
| TG(18:2/20:4/22:6) | 1.018 | 0.026450 | 1.2131 | TG |
| TG(16:0e/22:0/22:4) | 1.055 | 0.009047 | 2.1966 | TG |
| TG(18:1/13:0/18:3) | 1.039 | 0.024940 | 1.7223 | TG |
| TG(18:0e/18:0/18:2) | 1.018 | 0.017580 | 1.2826 | TG |
| TG(18:0/20:4/22:6) | 1.026 | 0.023800 | 1.4432 | TG |
| TG(15:0/6:0/18:2) | 1.084 | 0.002583 | 2.7004 | TG |
| TG(20:4e/11:1/18:4) | 1.052 | 0.018720 | 2.0585 | TG |
| TG(18:0e/20:0/22:6) | 1.047 | 0.014010 | 1.9666 | TG |
| TG(16:0/22:5/22:6) | 1.028 | 0.026920 | 1.458 | TG |
| TG(18:0/13:0/18:3) | 1.037 | 0.026440 | 1.6831 | TG |
| TG(18:2/22:6/22:6) | 1.041 | 0.000487 | 1.9322 | TG |
| TG(18:0e/20:1/22:4) | 1.034 | 0.037580 | 1.6448 | TG |
| TG(16:0/10:4/20:5) | 1.013 | 0.049420 | 1.0722 | TG |
| TG(16:0/22:6/22:6) | 1.045 | 0.013330 | 1.8918 | TG |
| TG(16:0/18:3/22:6) | 1.031 | 0.002399 | 1.6944 | TG |
| TG(18:4/18:2/18:4) | 1.044 | 0.017060 | 1.9229 | TG |
| TG(11:0/6:0/16:0) | 1.052 | 0.002024 | 2.1158 | TG |
| TG(18:3/17:1/18:2) | 1.026 | 0.018190 | 1.4747 | TG |
| TG(20:4e/11:3/18:1) | 1.041 | 0.003524 | 1.99 | TG |
| TG(20:0/11:3/16:0) | 1.033 | 0.023450 | 1.5973 | TG |
| TG(16:0/12:1/22:6) | 1.141 | 0.042200 | 2.9691 | TG |
| TG(16:0/20:1/20:4) | 1.016 | 0.021540 | 1.1299 | TG |
| TG(6:0/11:2/20:5) | 1.060 | 0.012670 | 2.096 | TG |
| TG(8:0/13:0/18:1) | 1.085 | 0.029090 | 2.4778 | TG |
| TG(8:0/8:0/14:2) | 1.270 | 0.000179 | 4.3414 | TG |
| TG(4:0/11:2/18:1) | 1.074 | 0.037920 | 2.2886 | TG |
| TG(18:3e/11:1/22:4) | 1.040 | 0.041590 | 1.708 | TG |
| TG(18:1/11:2/18:2) | 1.009 | 0.003920 | 1.0208 | TG |
| TG(18:4/18:2/20:4) | 1.036 | 0.005751 | 1.7749 | TG |
| TG(16:1/10:1/18:1) | 1.113 | 0.001296 | 3.0883 | TG |
| TG(14:0e/22:5/23:0) | 1.042 | 0.040320 | 1.7381 | TG |
| TG(4:0/11:3/18:4) | 1.066 | 0.030470 | 2.0089 | TG |
| TG(16:0/10:4/10:4) | 1.159 | 0.043710 | 3.1629 | TG |
| TG(17:0/6:0/18:2) | 1.132 | 0.005503 | 3.0466 | TG |
| TG(18:1/10:2/13:0) | 1.075 | 0.004338 | 2.4788 | TG |
| TG(22:6/11:2/12:4) | 1.035 | 0.004961 | 1.7147 | TG |
| TG(16:0/10:3/20:4) | 1.089 | 0.008346 | 2.7056 | TG |
| TG(16:0/10:3/21:0) | 1.026 | 0.040090 | 1.3947 | TG |
| TG(19:1/12:2/16:0) | 1.032 | 0.022070 | 1.6403 | TG |
| TG(16:0/10:0/22:6) | 1.094 | 0.043850 | 2.4396 | TG |
| TG(18:0e/11:2/20:5) | 1.041 | 0.032310 | 1.7147 | TG |
| TG(16:0/14:2/22:6) | 1.038 | 0.002818 | 1.835 | TG |
| TG(16:0/20:5/22:1) | 1.044 | 0.002983 | 2.0154 | TG |
| TG(18:0/20:4/24:2) | 1.039 | 0.026980 | 1.7178 | TG |
| TG(20:5/21:1/22:2) | 1.042 | 0.030590 | 1.7268 | TG |
| WE(3:0/18:1) | 1.070 | 0.036810 | 2.1943 | WE |
| ZyE(11:0) | 1.022 | 0.022550 | 1.343 | ZyE |

Table S3 KEGG Enrichment statistics

| Num | First Category | Second Category | Pathway Desciption | Pathway_ID |
| --- | --- | --- | --- | --- |
| 2 | Organismal Systems | Endocrine system | Oxytocin signaling pathway | map04921 |
| 2 | Organismal Systems | Endocrine system | Insulin secretion | map04911 |
| 2 | Organismal Systems | Nervous system | Dopaminergic synapse | map04728 |
| 2 | Organismal Systems | Nervous system | Cholinergic synapse | map04725 |
| 2 | Organismal Systems | Immune system | Neutrophil extracellular trap formation | map04613 |
| 2 | Organismal Systems | Digestive system | Cholesterol metabolism | map04979 |
| 2 | Human Diseases | Infectious disease: viral | Coronavirus disease - COVID-19 | map05171 |
| 2 | Organismal Systems | Circulatory system | Adrenergic signaling in cardiomyocytes | map04261 |
| 2 | Environmental Information Processing | Signal transduction | Sphingolipid signaling pathway | map04071 |
| 2 | Environmental Information Processing | Signal transduction | HIF-1 signaling pathway | map04066 |
| 2 | Organismal Systems | Digestive system | Pancreatic secretion | map04972 |
| 2 | Human Diseases | Cancer: specific types | Hepatocellular carcinoma | map05225 |
| 2 | Human Diseases | Infectious disease: bacterial | Shigellosis | map05131 |
| 2 | Human Diseases | Cardiovascular disease | Lipid and atherosclerosis | map05417 |
| 2 | Organismal Systems | Digestive system | Gastric acid secretion | map04971 |
| 2 | Organismal Systems | Immune system | Platelet activation | map04611 |
| 2 | Environmental Information Processing | Signal transduction | Apelin signaling pathway | map04371 |
| 2 | Organismal Systems | Endocrine system | GnRH secretion | map04929 |
| 2 | Human Diseases | Infectious disease: bacterial | Vibrio cholerae infection | map05110 |
| 2 | Organismal Systems | Nervous system | Long-term depression | map04730 |
| 2 | Human Diseases | Endocrine and metabolic disease | AGE-RAGE signaling pathway in diabetic complications | map04933 |
| 2 | Organismal Systems | Environmental adaptation | Circadian entrainment | map04713 |
| 2 | Environmental Information Processing | Signal transduction | Phospholipase D signaling pathway | map04072 |
| 2 | Organismal Systems | Endocrine system | Parathyroid hormone synthesis, secretion and action | map04928 |
| 2 | Organismal Systems | Sensory system | Phototransduction - fly | map04745 |
| 2 | Organismal Systems | Immune system | C-type lectin receptor signaling pathway | map04625 |
| 2 | Organismal Systems | Endocrine system | Thyroid hormone signaling pathway | map04919 |
| 2 | Human Diseases | Cancer: overview | Choline metabolism in cancer | map05231 |
| 2 | Cellular Processes | Cellular community - eukaryotes | Gap junction | map04540 |
| 2 | Organismal Systems | Immune system | Fc epsilon RI signaling pathway | map04664 |
| 2 | Environmental Information Processing | Signal transduction | Calcium signaling pathway | map04020 |
| 2 | Organismal Systems | Circulatory system | Vascular smooth muscle contraction | map04270 |
| 2 | Human Diseases | Neurodegenerative disease | Spinocerebellar ataxia | map05017 |
| 2 | Organismal Systems | Development and regeneration | Axon regeneration | map04361 |
| 2 | Environmental Information Processing | Signal transduction | Ras signaling pathway | map04014 |
| 2 | Organismal Systems | Nervous system | Long-term potentiation | map04720 |
| 2 | Organismal Systems | Excretory system | Endocrine and other factor-regulated calcium reabsorption | map04961 |
| 2 | Organismal Systems | Endocrine system | Adipocytokine signaling pathway | map04920 |
| 2 | Organismal Systems | Endocrine system | Estrogen signaling pathway | map04915 |
| 2 | Human Diseases | Infectious disease: parasitic | African trypanosomiasis | map05143 |
| 2 | Organismal Systems | Nervous system | Glutamatergic synapse | map04724 |
| 2 | Organismal Systems | Immune system | Fc gamma R-mediated phagocytosis | map04666 |
| 2 | Organismal Systems | Digestive system | Salivary secretion | map04970 |
| 2 | Human Diseases | Infectious disease: viral | Human cytomegalovirus infection | map05163 |
| 2 | Environmental Information Processing | Signal transduction | VEGF signaling pathway | map04370 |
| 2 | Organismal Systems | Endocrine system | GnRH signaling pathway | map04912 |
| 2 | Organismal Systems | Endocrine system | Melanogenesis | map04916 |
| 2 | Human Diseases | Infectious disease: parasitic | Leishmaniasis | map05140 |
| 2 | Human Diseases | Cancer: specific types | Non-small cell lung cancer | map05223 |
| 2 | Organismal Systems | Endocrine system | Relaxin signaling pathway | map04926 |
| 2 | Organismal Systems | Nervous system | Retrograde endocannabinoid signaling | map04723 |
| 2 | Human Diseases | Infectious disease: viral | Kaposi sarcoma-associated herpesvirus infection | map05167 |
| 2 | Human Diseases | Infectious disease: viral | Human immunodeficiency virus 1 infection | map05170 |
| 2 | Environmental Information Processing | Signal transduction | Rap1 signaling pathway | map04015 |
| 2 | Environmental Information Processing | Signal transduction | MAPK signaling pathway | map04010 |
| 2 | Organismal Systems | Nervous system | Neurotrophin signaling pathway | map04722 |
| 2 | Organismal Systems | Immune system | Chemokine signaling pathway | map04062 |
| 2 | Cellular Processes | Transport and catabolism | Efferocytosis | map04148 |
| 2 | Organismal Systems | Endocrine system | Thyroid hormone synthesis | map04918 |
| 2 | Organismal Systems | Endocrine system | Aldosterone synthesis and secretion | map04925 |
| 2 | Environmental Information Processing | Signal transduction | cAMP signaling pathway | map04024 |
| 2 | Human Diseases | Neurodegenerative disease | Parkinson disease | map05012 |
| 2 | Human Diseases | Drug resistance: antineoplastic | EGFR tyrosine kinase inhibitor resistance | map01521 |
| 2 | Organismal Systems | Digestive system | Carbohydrate digestion and absorption | map04973 |
| 2 | Human Diseases | Cancer: overview | Pathways in cancer | map05200 |
| 2 | Human Diseases | Neurodegenerative disease | Pathways of neurodegeneration - multiple diseases | map05022 |
| 2 | Human Diseases | Cancer: specific types | Glioma | map05214 |
| 2 | Organismal Systems | Immune system | Natural killer cell mediated cytotoxicity | map04650 |
| 2 | Human Diseases | Cancer: overview | PD-L1 expression and PD-1 checkpoint pathway in cancer | map05235 |
| 2 | Organismal Systems | Immune system | Th17 cell differentiation | map04659 |
| 2 | Environmental Information Processing | Signal transduction | ErbB signaling pathway | map04012 |
| 2 | Organismal Systems | Immune system | B cell receptor signaling pathway | map04662 |
| 2 | Organismal Systems | Immune system | T cell receptor signaling pathway | map04660 |
| 2 | Organismal Systems | Endocrine system | Growth hormone synthesis, secretion and action | map04935 |
| 2 | Organismal Systems | Sensory system | Inflammatory mediator regulation of TRP channels | map04750 |
| 2 | Organismal Systems | Immune system | Th1 and Th2 cell differentiation | map04658 |
| 2 | Human Diseases | Immune disease | Systemic lupus erythematosus | map05322 |
| 2 | Environmental Information Processing | Signal transduction | NF-kappa B signaling pathway | map04064 |
| 2 | Metabolism | Lipid metabolism | Glycerolipid metabolism | map00561 |
| 2 | Human Diseases | Cardiovascular disease | Diabetic cardiomyopathy | map05415 |
| 2 | Organismal Systems | Nervous system | Serotonergic synapse | map04726 |
| 2 | Organismal Systems | Digestive system | Vitamin digestion and absorption | map04977 |
| 2 | Metabolism | Amino acid metabolism | Glycine, serine and threonine metabolism | map00260 |
| 2 | Metabolism | Lipid metabolism | Glycerophospholipid metabolism | map00564 |
| 4 | Organismal Systems | Endocrine system | Regulation of lipolysis in adipocytes | map04923 |
| 4 | Human Diseases | Endocrine and metabolic disease | Insulin resistance | map04931 |
| 4 | Organismal Systems | Environmental adaptation | Thermogenesis | map04714 |
| 4 | Human Diseases | Infectious disease: parasitic | Amoebiasis | map05146 |
| 4 | Organismal Systems | Digestive system | Fat digestion and absorption | map04975 |

1. Number：number of metabolites enriched in this pathway; (2) Description：KEGG pathway name description; (3) Pathway ID：KEGG pathway ID.
